# The biotechnological importance of the plant-specific NAC transcription factor family in crop improvement

**DOI:** 10.1007/s10265-021-01270-y

**Published:** 2021-02-22

**Authors:** Sadhana Singh, Hiroyuki Koyama, Kaushal K. Bhati, Anshu Alok

**Affiliations:** 1 International Crops Research Institute for the Semi-Arid Tropics (ICRISAT), Patancheru, India; 2 Laboratory of Plant Cell Technology, Faculty of Applied Biological Sciences, Gifu University, Gifu, 501-1193 Japan; 3 Louvain Institute of Biomolecular Sciences, Catholic University of Louvain, Louvain-la-Neuve, Belgium; 4 Department of Biotechnology, UIET, Punjab University, Chandigarh, India

**Keywords:** Arabidopsis, Cereals, Legumes, NAC, Transcription factor

## Abstract

Climate change, malnutrition, and food insecurity are the inevitable challenges being faced by the agriculture sector today. Plants are susceptible to extreme temperatures during the crucial phases of flowering and seed development, and elevated carbon levels also lead to yield losses. Productivity is also affected by floods and droughts. Therefore, increasing plant yield and stress tolerance are the priorities to be met through novel biotechnological interventions. The contributions of *NAC* genes towards enhancing plant survivability under stress is well known. Here we focus on the potential of *NAC* genes in the regulation of abiotic stress tolerance, secondary cell wall synthesis, lateral root development, yield potential, seed size and biomass, ROS signaling, leaf senescence, and programmed cell death. Once naturally tolerant candidate *NAC* genes have been identified, and the nature of their association with growth and fitness against multi-environmental stresses has been determined, they can be exploited for building inherent tolerance in future crops via transgenic technologies. An update on the latest developments is provided in this review, which summarizes the current understanding of the roles of *NAC* in the establishment of various stress-adaptive mechanisms in model and food crop plants.

## Introduction

Transcription factors (TFs) contribute to about 7% of the coding part of plant transcriptomes. Several TFs also function as immediate or early stress-responsive factors against biological triggers (Hoang et al. 2017; Lindemose et al. 2013). Transcription factors are well known to initiate the reaction cascades by binding to *cis*-elements upstream of target genes, which encode proteins for particular biological roles (Baillo et al. 2019). A typical TF is comprised of four parts: a conserved DNA-binding part, a variable transcription-regulation part, an oligomerization part, and a nuclear localization signal (NLS) for protein import into the nucleus. The preferential involvement of some TFs as master regulators of signaling and regulatory mechanisms in stress acclimatization is well explored (Hoang et al. 2019). For instance, members of TF sub-families (CBF/DREB, MYB, WRKY, NAC, bZIP, APETALA, C2-H2 type zinc fingers, basic helix-loop-helix etc.) are recognized to be intimately involved in transforming stress signals into alterations in gene expression and thereby triggering adaptive responses in plant cells (Kosová et al. 2015; Lata et al. 2011). There are several recent examples of stress-related TFs from the NAC (NAM, ATAF, and CUC) TF subfamily. The acronym NAC originates from three different genes (*No Apical Meristem*: *NAM*, *Arabidopsis Transcription Activation Factor*: *ATAF*, and *Cup*-*Shaped Cotyledon*: *CUC*), where NAC domain was first reported (Aida 1997; Sablowski et al.^1998^). NAC is considered one of the largest TF families in plants, with more than 100 genes reported in *Arabidopsis thaliana* (L.) Heynh. (Arabidopsis) as well as several other members of the plant kingdom (Table 1) (Baillo et al. 2019; Singh et al. 2019).

**Table 1:** *NAC* gene distribution among common cereal and legume crops (acc. to Plant Transcription Factor Database version 4)

Species name	No of NAC genes
Birdsfoot trefoil (*Lotus japonicus*)	116
Barrel medic (*Medicago truncatula*)	123
Soybean (*Glycine soja*)	173
Soybean (*Glycine max)*	269
Pigeonpea (*Cajanus cajan*)	96
Peanut (*Arachis hypogaea*)	32
Peanut (*Arachis ipaensis*)	83
Peanut (*Arachis duranensis*)	82
Chickpea (*Cicer arietinum*)	96
Common bean *(Phaseolus vulgaris)*	106
Mung bean (*Vigna radiata*)	82
Cowpea (*Vigna unguiculata*)	20
Rice (*Oryza sativa* -japonica)	170
Rice (*Oryza sativa* -indica)	158
Maize (*Zea mays*)	189
Barley (*Hordeum vulgare*)	150
Foxtail millet (*Setaria italica*)	165
Sorghum (*Sorghum bicolor*)	180
Wheat (*Triticum aestivum*)	263
Pearl millet *(Pennisetum glaucum)*	151

The NAC TFs control plant development, senescence, morphogenesis, and abiotic stress tolerances (Kosová et al. 2015; Singh et al. 2016). It is noteworthy that NAC members constitute a well conserved DNA binding (NAC) domain at the N-terminal and a varied C-terminal domain that generally has an intrinsically disordered region. The intrinsically disordered region contributes to various biological functions among sub-families, for instance, regulates transcription via an ABA-dependent or ABA-independent pathway to modulate stress-related gene expression (Baillo et al. 2019; Ernst et al. 2004). There are even more complex functions associated with NACs, for example, influencing miRNA mediated cleavage of mRNAs (Mallory et al. 2004) and ubiquitin-dependent proteolysis (Xie et al. 2002). Whether this complex TF family has roles limited to stress and plant development, is still under debate.

**Fig. 1:**
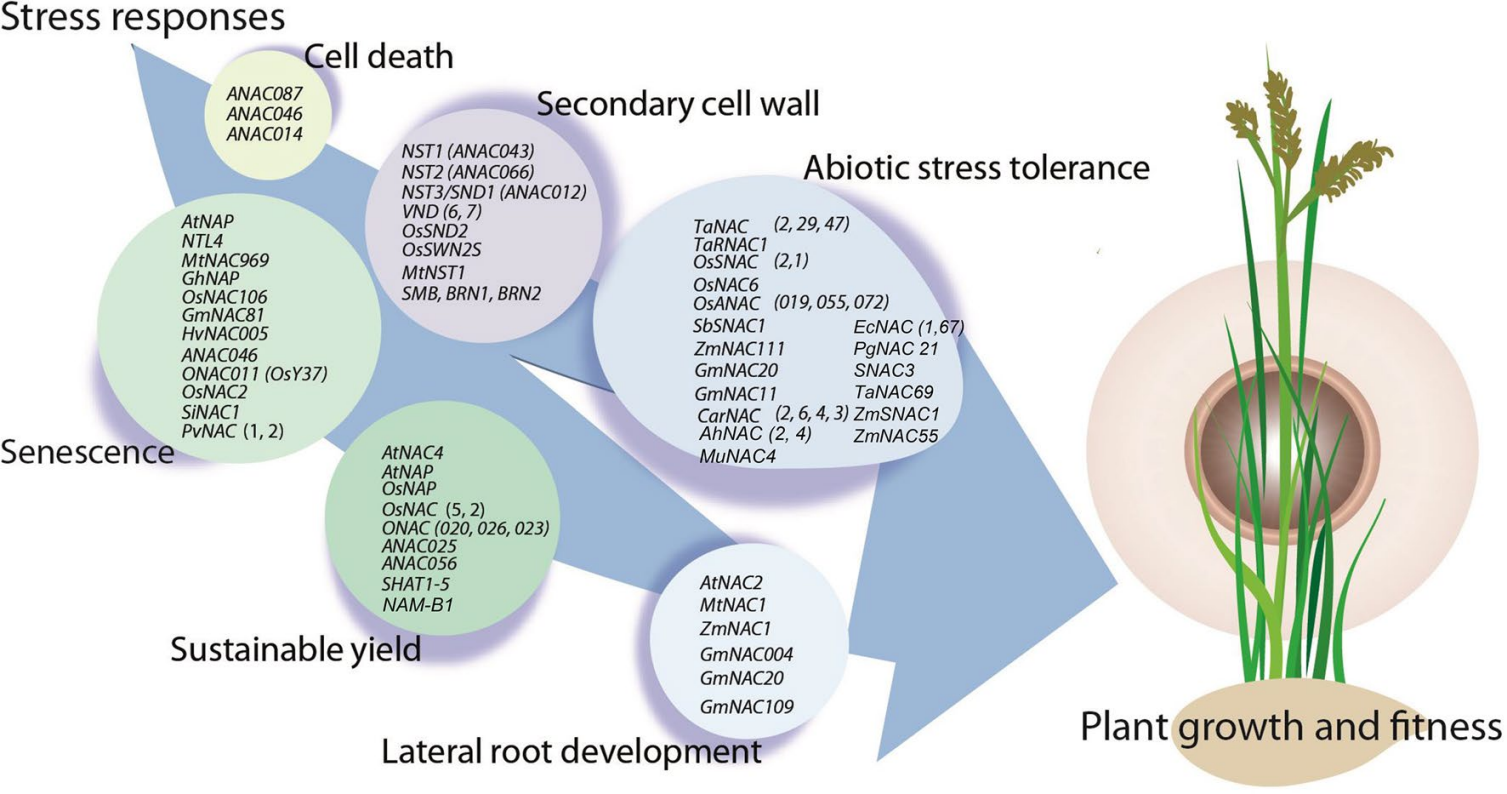
Diagrammatic representation of potential *NAC* genes involved in the primary and secondary phases of plant growth and adaptive response

This review primarily focuses on the role this plant-specific TF subfamily plays during general developmental and adaptive regulation in food crops. Here, we summarize the evidence currently available (particularly from the past decade), concerning the range of NAC functions in controlling the growth and development of the plants, and their participation in the plant’s adaptive response against a variety of stresses (Fig. 1). We also discuss important translational aspects associated with NAC TFs in combating the latest challenges of climate change through biotechnological interventions.

## NAC proteins: structure, function, regulation co-relations

As noted above, NAC proteins are represented by a conserved N-terminal DNA binding domain and a variable transcription regulatory region at the C-terminus, which plays a role in either transcriptional activation or repression of stress-induced genes and pathways (Puranik et al. 2012; Yamaguchi et al. 2010, Fig. 2). The N-terminus is separated into sub-domains dubbed A through E. Within the conserved N-terminal domain too, the sequence varies. Sub-domains C and D are highly conserved and positively charged. These sub-domains bind to DNA. The positions Lys123 and Lys126 (β4–5; sub-domain D), Val119–Ser183 (β4–6; sub-domain D–E), and Lys79, Arg85, and Arg88 (β1–2; sub-domain C) are considered essential residues for DNA binding (Chen et al. 2011; Ernst et al. 2004). Among these, Arg88 has so far been found conserved in all NAC proteins (Puranik et al. 2012). The redundancy of Lys79 and Arg85 has also been proposed as a reason for the varying DNA binding abilities of NAC proteins (Jensen et al. 2010). The nuclear localization signal rests in the sub-domain D, mediated by the lysine residues which help in nuclear shuttling (Le et al. 2011; Olsen et al. 2005a, b; Tran et al. 2009). Sub-domains B and E are relatively divergent and may be contributing to NAC protein function diversity, along with the C-terminal domains. Sub-domain A plays a role in functional dimer formation through Leu14–Thr23 and Glu26–Tyr31 residues (Ernst et al. 2004; Jensen et al. 2010; Olsen et al. 2005a; Ooka et al. 2003; Puranik et al. 2012). Model of a typical NAC protein from pearl millet (PgNAC21) showing α-helix and twirled β-sheet bound to target DNA can be seen in Fig. 3a, b.

**Fig. 2:**
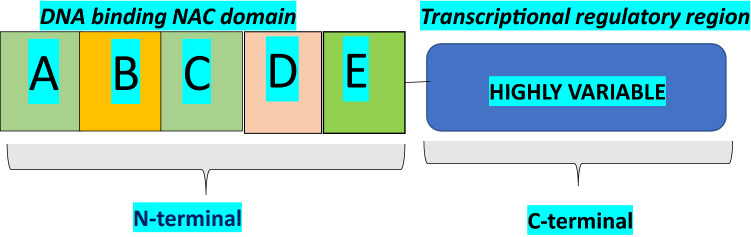
A conceptual diagram of a typical NAC protein showing N- and C-terminals

**Fig. 3:**
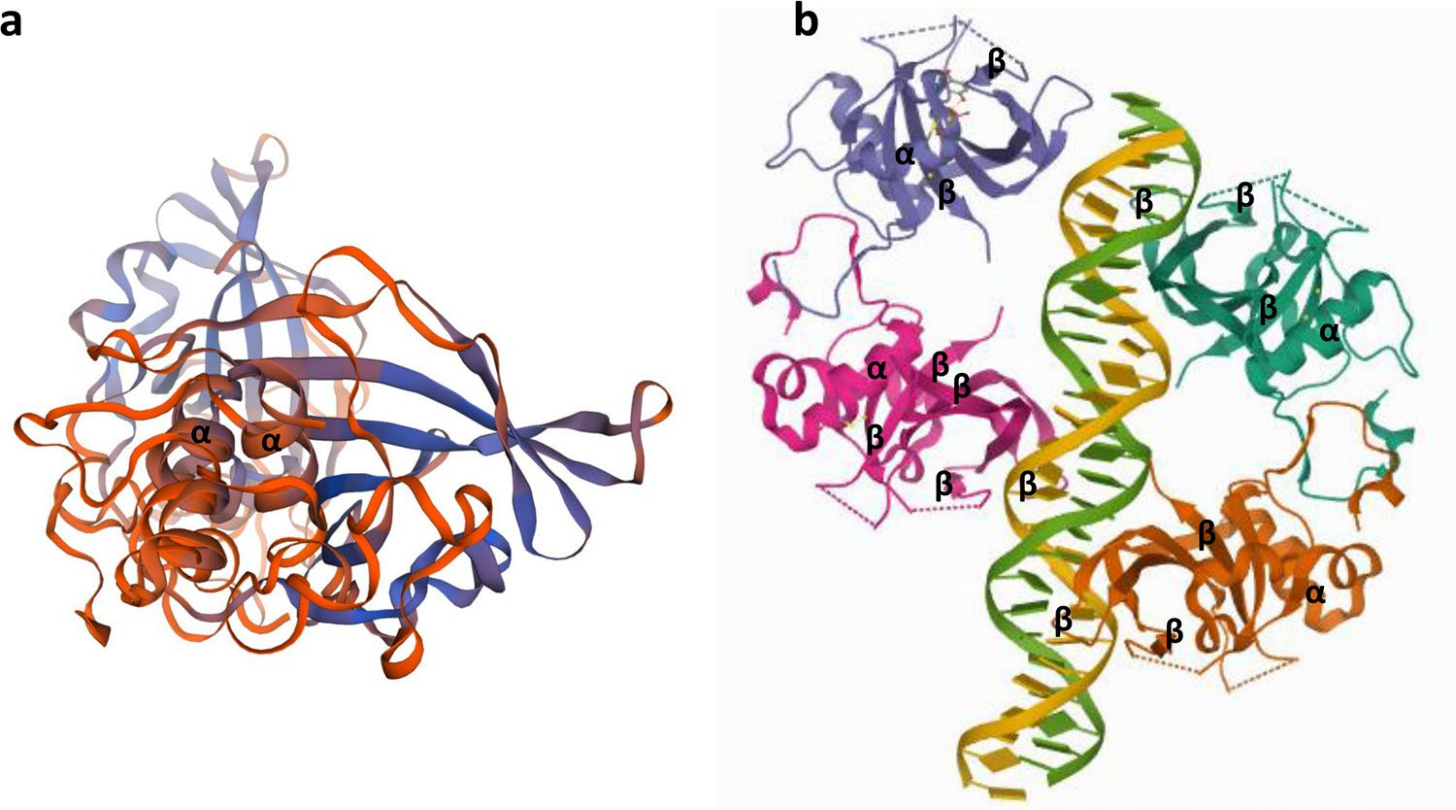
Representation of 3-D structure of NAC protein (PgNAC21) from pearl millet. **a** Structure prediction of PgNAC21 protein by homology modelling server using SWISS-MODEL. **b** PgNAC21 structure depiction by PDB (Protein Data Bank) showing α-helix and antiparallel β-sheet for DNA binding

The NAC domain has also been implicated in protein binding activities, which in turn may be crucial to various functions of the NAC proteins including stress tolerance (Olsen et al. 2005a; Tran et al. 2007; Yamaguchi et al. 2010). The C-terminal domain has a low complexity region containing serine–threonine, proline–glutamine, or acidic residue repeats (Fang et al. 2008). This causes an intrinsic disorder and renders a lack of stable three-dimensional structures (Jensen et al. 2010). However, this makes the NACs interact with diverse targets (*Lipoxygenase*, *DEAD/DEAH box helicase*, *Pectin methyl esterase inhibitor*, and *Homeobox associated proteins* coding genes, etc.) (Grover et al. 2014). These motifs are conserved within a given sub-family but vary among groups of sub-families.

To this end, there are several sub-families of the NAC proteins, classified and described in the literature (Hu et al. 2010; Lv et al. 2016). Some works reported as many as 18 sub-families for NAC TFs (Shang et al. 2013), though no definitive role has been assigned to a particular sub-family. These uncertainties emerge because of the rich diversity of NAC domains. This is also the reason for limited success in identifying the role of *NAC* individual genes within a given sub-family (Puranik et al. 2012). Additionally, these attributes of NAC sub-families are also indicative of redundancy for the target gene members. However, identifying the expression pattern will be the first step in understanding how these NAC members are involved in stress response.

## Expression of NAC genes during development and stress response

Stress-responsive *NAC* genes are expressed differentially and are highly regulated at the transcriptional level. Phylogenetic analysis indicates that stress-responsive NAC TFs contain the closely homologous NAC domain (Fang et al. 2008; Tran et al. 2009). Analysis of Arabidopsis seedlings using the Agilent 22 K Oligo DNA microarray revealed that 12 *ANAC* genes out of 67 (reported in the genome), were induced by ABA and abiotic stresses (Fujita et al. 2004). Arabidopsis salt-stressed root transcriptome using 70mer oligomer microarray probes comprising 23,686 genes, exposed differential expression in the *ANAC* genes: 23 were up-regulated whereas seven genes were suppressed (Jiang and Deyholos 2006). Other work by Matsui et al. (2008) showed that, when the whole genome expression profiling (tiling array) in three-week old Arabidopsis seedlings was subjected to drought, cold, salinity, and ABA stress, 30 *ANAC* genes out of 108, were up-regulated under at least one of the imposed stresses. Later, Jiang et al. (2014) reported hyper-sensitivity of lines of Arabidopsis overexpressing the *RhNAC3* gene from rose (*Rosa hybrida*) upon ABA or drought stresses during seed germination and leaf closure stages. Nevertheless, NAC family members exhibit preferential gene expression at various life cycle stages, or during the development of tissues. In this regard, NTM1-Like or “NAC with Transmembrane Motif 1”-Like (NTLs), a class of membrane-associated NAC transcription factor, which is known to be associated with transcriptional response to external stimuli, and linked to delayed flowering (*NTL8*). Investigations revealed that higher expression of NTLs was linked to a reduced expression of *FLOWERING LOCUS T* (*FT*). This in turn resulted in flowering delay, reduced growth and leaf curling in Arabidopsis (Kim et al. 2007).

In *Medicago truncatula*, out of 97 *MtNAC* candidate genes, 40 *NACs* were expressed in different tissues –roots, buds, seed pods, and flowers (Ling et al. 2017). Among the expressed NACs, nine genes were preferentially expressed in roots, 13 in seed pods, and three in buds. Moreover, RNA-seq data analysis showed that 44 *MtNAC* genes were found regulated by various stresses such as cold, drought, salt, freezing, and ABA-stress. Of these, 17 *MtNAC* genes were up-regulated, whereas only *MtNAC1* was down-regulated under all stresses. Further, 33 genes were induced exclusively by cold and drought, whereas 12 genes were specifically expressed during freezing and salinity stresses. Expression of *MtNAC50* was highly up-regulated during cold, and *MtNAC95* was up-regulated in salt, drought, and ABA stresses. Similarly, *MtNAC57* and *MtNAC73* were up-regulated during all stresses apart from freezing (Ling et al. 2017).

Fang and co-authors (Fang et al. 2008) employed 70mer oligomer microarray analysis to identify 140 putative ONAC-like TFs in rice (*Oryza sativa*). Twenty-one of these were induced by drought or salinity and five were repressed by stress in the seedling stage. The same authors (Fang et al. 2008) validated 20 *ONAC* genes with elevated expression levels using rice seedlings and found that five genes were induced by dehydration, 19 by salt, and 16 by cold.

Ha et al. (2014) used phylogenetic analysis to identify 71 *CaNAC* genes, including eight membrane-bound NACs, from the chickpea (*Cicer arietinum*) genome. Nineteen of the predicted 23 dehydration-related *CaNAC* genes were specific to either roots or leaves. Fourteen genes were up-regulated, whereas four were down-regulated under dehydration stress in leaves. *CaNAC06* and *CaNAC67* were the most up-regulated genes with 200 to 300-fold. The highly down-regulated genes were *CaNAC02* and *CaNAC04*. In root tissues, 12 genes were up-regulated, and three genes were down-regulated during dehydration. By comparison, 88 *NAC* genes were identified in the pigeon pea (*Cajanus cajan*), using homology searches, and de novo approaches based on the published pigeon pea draft genome (Satheesh et al. 2014). Of these, 36 *NAC* genes were identified as putatively drought-responsive, based on the phylogenetic analysis (Satheesh et al. 2014). Several stress-responsive cis-acting regulatory elements (MYB, TC rich repeats, HSE element, ABRE element) were reported from promoter regions of these *NAC* genes, which may contribute to enhancing the stress tolerance.

More recently, Hussain et al. (2017) identified 139 *GmNAC* genes in the soybean (*Glycine max*), and observed genotype-based *GmNAC* gene expression in response to drought. Out of these 139 *GmNAC* genes, 28 genes were predicted to be drought-responsive, based on the phylogenetic analysis. Eight of the *GmNAC* genes (*GmNAC004*, *021*, *065*, *066*, *073*, *082*, *083*, *08*) showed higher expression levels in drought-resistant cultivars than in drought-sensitive cultivars and were induced despite the level of dehydration sensitivity of cultivars. Earlier, Le et al. (2011) reported 50 putative stress-responsive *GmNAC* genes, based on the sequence alignment and phylogenetic analysis with known Arabidopsis (*ANAC055*, *ANAC072*, *ANAC019*) and rice (*SNAC1/SNAC2*) stress-responsive *NAC* genes. Sixteen *GmNACs* were tissue-specific and highly expressed in roots and flowers. Twenty-five *GmNACs* were induced, and six were repressed by two-fold or more under dehydration stress in roots and shoots of soybean. *GmNAC085*, which is identical to the widely-studied *SNAC1/ONAC2*, displayed induction of 390-fold in shoots and 20-fold in roots and was amongst those genes most highly expressed gene during dehydration.

**Fig. 4:**
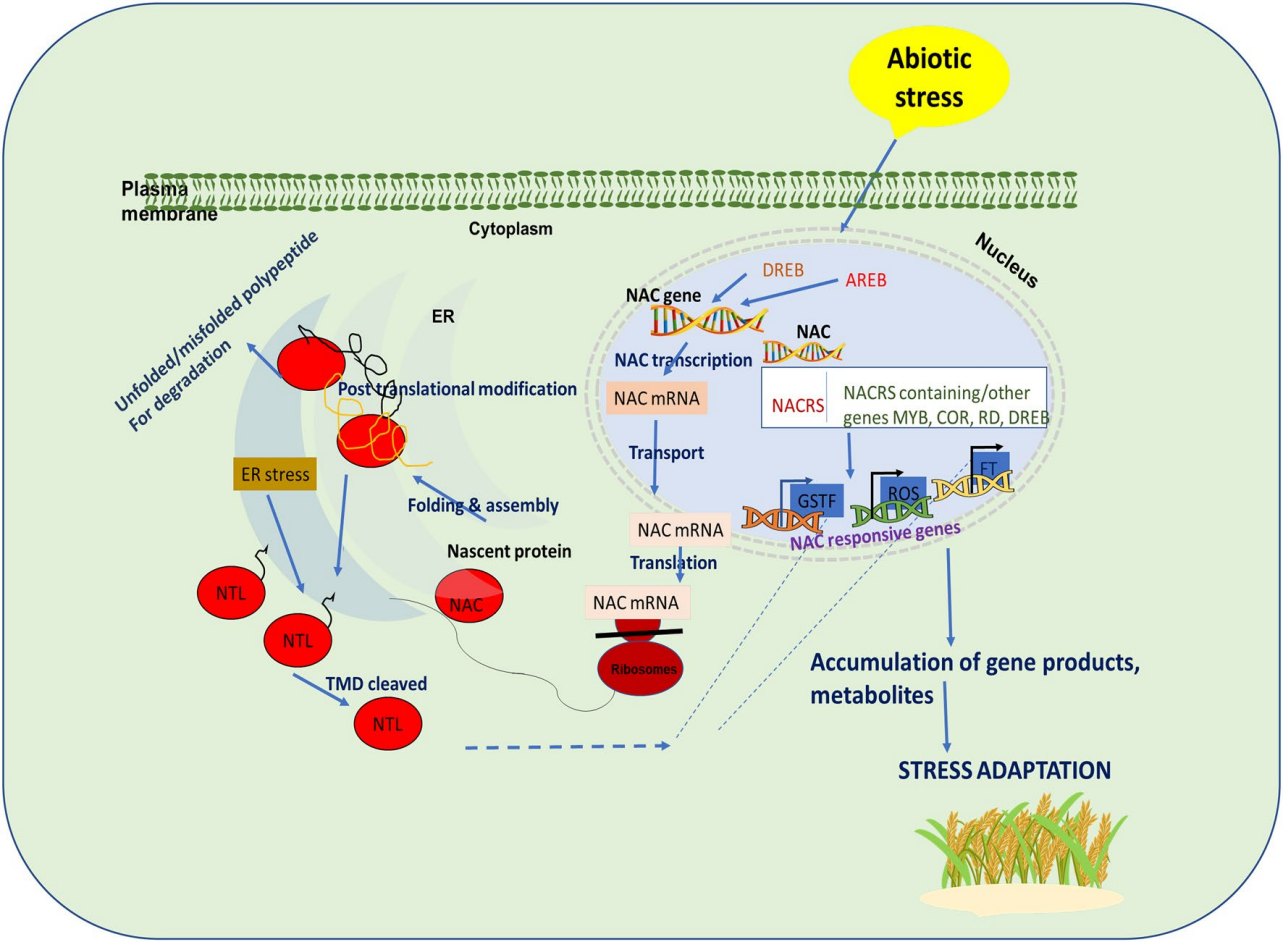
Schematic representation of NAC regulation in plant abiotic stress tolerance. Upon encountering stress, the NAC gene is induced by ABA dependent/independent pathway, which in turn binds to the promoter sequences of other genes (*COR*, *DREB* etc.), thereby regulating ROS, GSTF, FT expression. On the other hand, accumulation of unfolded/misfolded proteins triggers unfolded protein response (UPR) in the ER. Thus, membrane bound NAC domain proteins (NTLs) are activated by ER stress and undergo conformational changes (TMD cleavage) to modulate the expression of stress-induced gene. NTLs thus, plays important role in communicating ER stress signaling from PM to nucleus to mitigate the ER stress. *NACRS* NAC recognition site/sequence, *COR* Cold regulated, *RD* responsive to desiccation, *ER* endoplasmic reticulum, *NTL* NAC with Transmembrane Motif 1″-Like, *TMD* transmembrane domain, *AREB* abscisic acid-responsive element-binding protein, *DREB* drought-responsive element-binding, *ROS* reactive oxygen species, *GSTF* glutathione S-transferase; *FT* flowering locus T; *UPR* unfolded protein response, *PM* plasma membrane

**Fig. 5:**
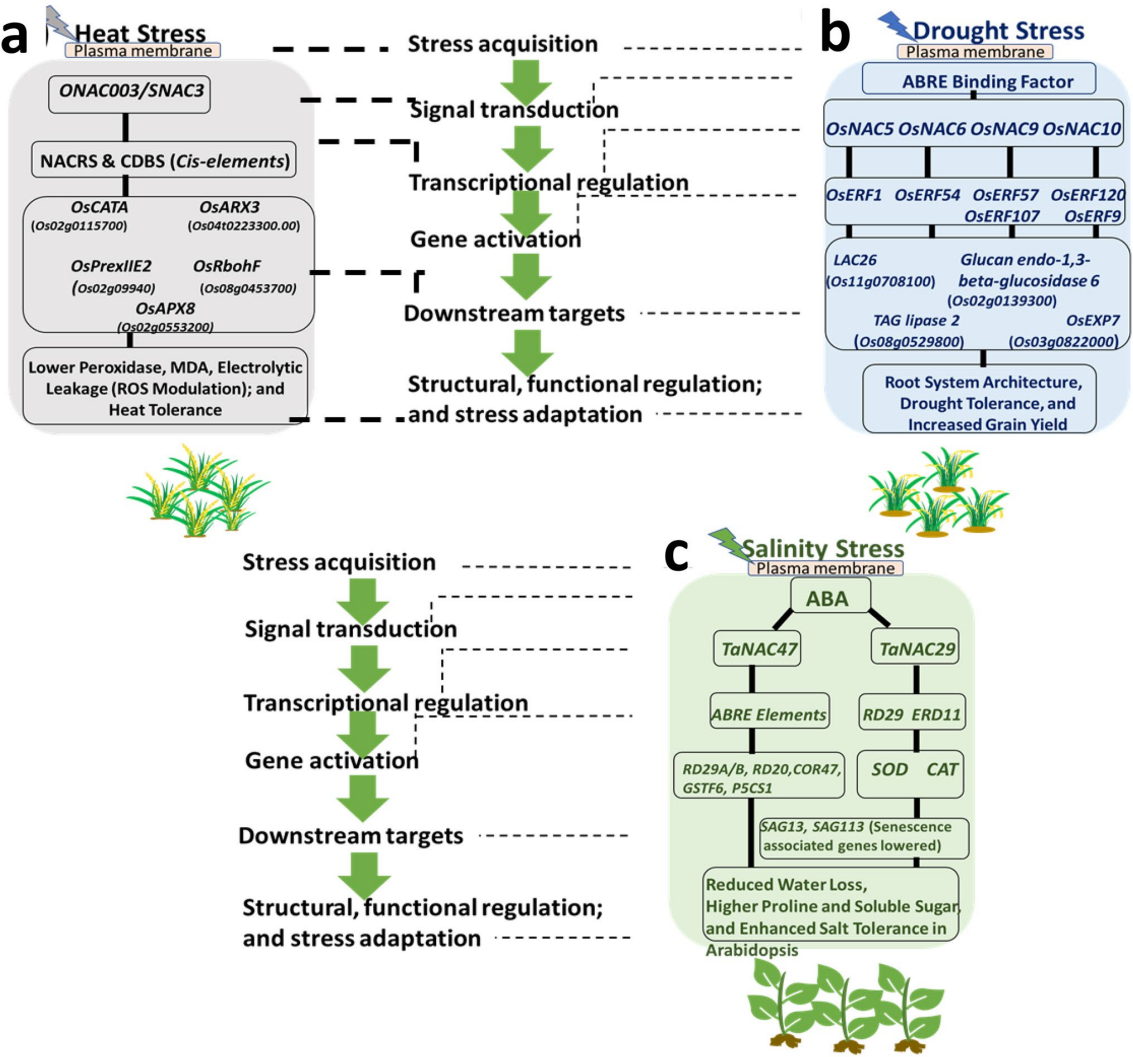
Representation of the NAC transcriptional regulatory network associated with stress adaptation from case studies of rice (Drought: Chung et al. 2018, Heat: Fang et al. 2015) and wheat (Salinity: Zhang et al. 2016, Huang et al. 2015). **a** During heat, *ONAC003* (*SNAC3*) expression is induced upon perceiving stress. Binding of *ONAC003* with NAC-specific NACRS (NAC recognition sequences/site) and CDBS (Core DNA-binding sequence,) and is activated. This results in up-regulation of targets *CATA* (*Catalase isozyme A*-*like*), *APX3 (Ascorbate peroxidase 3*), *APX8* (*Ascorbate peroxidase 8)*, *RbohF* (*NADPH oxidase*), *and Prx IIE2* (*Peroxiredoxin*), thereby reducing peroxide levels (H_2_O_2_), MDA (Malondialdehyde) and electrolytic leakage. This modulation of ROS metabolism by target gene up-regulation results in heat tolerance in rice plants. **b** *OsNAC* regulatory networks include the activation of *OsNAC5*, *6*, *9*, and *10* TFs during drought stress. Further, *OsNAC* activation leads to the up-regulation of various ERF (Ethylene responsive factor) domain genes. Target genes, such as *OsERF1*, *OsERF54*, *OsERF57* and *OsERF107*, and *OsERF9* are directly up-regulated by *OsNAC5*, *OsNAC6*, *OsNAC9* and *OsNAC10.* The up-regulation of target genes (*OsERF1*, *OsERF54*, *OsERF57*, *OsERF107*, and *OsERF9*) leads to the increased expression of *LAC26* (*Laccase*-*22*-*like)*, *TAG lipase 2* (*Triacylglycerol lipase 2)*, *Glucan endo*-*1*,*3*-*beta*-*glucosidase 6*, and *OsEXP7 (expansin*-*A7*-*like*), which altogether resulted in alteration of the root architectures for drought tolerance and enhanced grain yield in rice. **c** In case of salinity stress, *TaNAC47* and *TaNAC29* expression is induced in response to stress signaling via the ABA dependent pathway. *TaNAC47* binds to ABRE cis-elements and activates transcription. This leads to up-regulation of targets such as, *RD29A (Responsive to desiccation A)*, *RD29B* (*Responsive to desiccation B)*, *GSTF6* (*Glutathione S*-*transferase F6)*, *RD20* (*Responsive to desiccation 20*), *P5CS1* (*Δ*^*1*^-*pyrroline*-*5*-*carboxylate synthetase 1*), *COR47* (*Cold regulated 47*) in Arabidopsis. This, in turn, leads to reduced water loss and increased proline and soluble sugar, further improving survival under salinity stress in plants. Similarly, increased expression of *TaNAC29* during salinity stress leads to up-regulation of *RD29b* (*Responsive to desiccation 29b*) and *ERD11 (Early responsive to dehydration)* target genes, thereby enhancing SOD (Superoxide dismutase) and CAT (Catalase) activity, resulting in reduced water and enhanced salinity tolerance in Arabidopsis

**Fig. 6:**
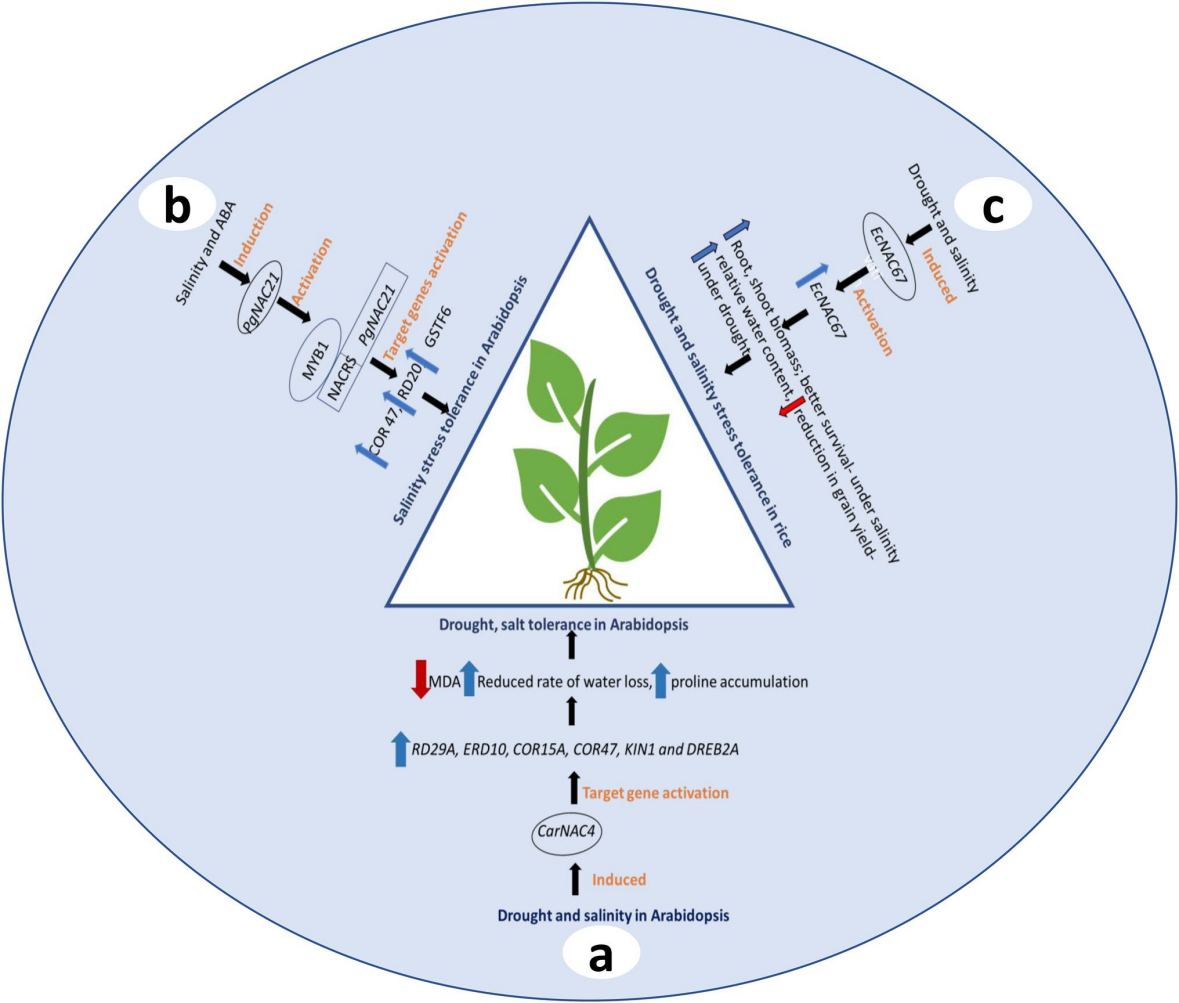
Representation of the NAC transcriptional regulatory network associated with stress adaptation from cases studied in SAT cereals (Shinde et al. 2019) and legume (Yu et al. 2016) to provide stress tolerance in Arabidopsis and rice plants. Blue arrows indicate up-regulation; red arrows indicate down-regulation. **a** Chickpea *CarNAC4* is induced under drought and salinity. Upon activation, *CarNAC4* in Arabidopsis plants causes up-regulation of *RD29*, *COR15A*, *KIN1*, *DREB* genes, resulting in lower MDA and low water loss. **b** The pearl millet *PgNAC21* regulatory network involves induction of *NAC21* in response to salt and ABA exposure. Binding of *MYB1* to upstream regulatory sequences (NACRS) of *PgNAC21* in Arabidopsis plants activates its expression, which then leads to up-regulation of target gene (*COR*, *RD20*, *GSTF6*), resulting in salt stress adaptation. **c** Finger millet *EcNAC67* is induced during salinity and drought stress. Activation of *EcNAC67* in rice plants causes a greater abundance of gene transcripts, which result in increased plant biomass, lower yield loss and better survival of rice plants under drought and salt conditions. *SAT* semi-arid tropics, *NACRS* NAC recognition site, *COR15A* cold regulated, *RD29* responsive to desiccation29, *GSTF6* glutathione S-transferase F6, *MDA* malondialdehyde

The overall expression patterns observed across different plants imply the selective up-regulation of individual NAC members and indicate the contribution of NAC TFs in the stress adaptation scheme (Figs. 4, 5, 6). However, there is still debate about whether there are several NAC candidates with conserved roles across plant species in response to stress type. In the next sections, we summarize the NAC roles more specific to various stress adaptive schemes and how this information could be utilized to exploit NACs for crop improvement.

## Potential of NACs in crop improvement

### Abiotic stress tolerance

Although NAC proteins were initially found to associate with plant development, their involvement in stress responses is now being increasingly recognized (Jensen and Skriver 2014). Classically, three closely associated NAC proteins members (ANA019, ANAC055, and ANAC072 (RD26)) respond to various abiotic stresses and hormones such as dehydration, freezing, salinity, wounding, jasmonic acid (JA), and abscisic acid (ABA). Most of the knowledge about these proteins stems from genome-wide and functional genomics studies in the most studied plants such as soybean (Hao et al. 2011), rice (Ohnishi et al. 2005), and Arabidopsis (Jiang et al. 2014), among others. Over-expression of these TFs has often provided substantial evidence supporting their activities (Table 2). For instance, transgenic rice plants overexpressing the *SNAC2* gene demonstrated a significantly enhanced germination and higher growth rates than the wild type (WT) under imposed salinity (Hu et al. 2008). Additionally, these plants showed greater vigor than non-transformed controls under a freezing environment. Similarly, transgenic tobacco lines overexpressing the poplar *NAC13* gene, demonstrated enhanced salt tolerance (Cheng et al. 2020).

**Table 2:** Evidence of attaining abiotic stress adaptation in NAC over-expressor plants specifically cereals and legumes

NAC TF in abiotic stress adaptation in plant	Source plant	Stress acquired in crop species	Method (functional validation)	Stress response	References
*AemNAC2*, *AemNAC3*	*Aegilops markgrafii* (wild relative of the cultivated wheat)	Wheat (cultivar-*Bobwhite*)	Over-expression	Cadmium (Cd) tolerance	Du et al. (2020)
*AhNAC2*	Peanut (*Arachis hypogaea*)	Arabidopsis	Over-expression	Drought and Salt stress tolerance	Liu et al. (2011)
*AhNAC3*	Peanut (*Arachis hypogaea*)	Tobacco *(Nicotiana tabacum*)	Over-expression	Dehydration and Drought tolerance by increasing superoxide scavenging	Liu et al. (2013)
*AhNAC4*	Peanut (*Arachis hypogaea*)	Tobacco	Over-expression	Drought tolerance	Tang et al. (2017)
*AtNAC2 (ANAC092)*	*Arabidopsis*	Peanut (*Arachis hypogaea*)	Over-expression	Drought and Salinity and improved yield under water-limited conditions	Patil et al. (2014)
*CarNAC4*	Chickpea (*Cicer arietinum*)	Arabidopsis	Over-expression	Drought and Salt stress tolerance	Yu et al. (2016)
*CarNAC6*	Chickpea (*Cicer arietinum*)	Arabidopsis	Over-expression	Dehydration tolerance and lateral root development	Liu et al. (2017)
*EcNAC1*	Finger millet (*Eleusine coracana*)	Tobacco	Over-expression	Water-deficit and Salt stress	Ramegowda et al. (2012)
*EcNAC67*	Finger millet (*Eleusine coracana*)	Rice	Over-expression	Salinity and Drought stress tolerance	Rahman et al. (2016)
*GmNAC019*	Soybean (*Glycine max*)	Arabidopsis	Over-expression	Drought tolerance	Hoang et al. (2019)
*GmNAC109*	Soybean (*Glycine max*)	Arabidopsis	Over-expression	Drought and Salt stress tolerance	Yang et al. (2019)
*GmNAC11*	Soybean (*Glycine max*)	Arabidopsis	Over-expression	Salt tolerance	Hao et al. (2011)
*GmNAC20*	Soybean (*Glycine max*)	Arabidopsis	Over-expression	Salinity and freezing tolerance	Hao et al. (2011)
*GmSNAC49*	Soybean (*Glycine max*)	Arabidopsis	Over-expression	Drought tolerance	So and Lee, (2019)
*HvSNAC1*	Barley (*Hordeum vulgare)*	Barley	Over-expression	Drought tolerance	Al Abdallat et al. (2014)
*MuNAC4*	Horse gram (*Macrotyloma uniflorum*)	Peanut	Over-expression	Drought tolerance	Pandurangaiah et al. (2014)
*ONAC022*	Rice (*Oryza sativa*)	Rice	Over-expression	Drought and Salt tolerance	Hong et al. (2016)
*ONAC066*	Rice (*Oryza sativa*)	Rice	RNAi	Drought and Oxidative stress	Yuan et al. (2019)
*OsNAC14*	Rice (*Oryza sativa*)	Rice	Over-expression	Drought tolerance (higher panicle number and filling rate)	Shim et al. (2018)
*ONAC095*	Rice (*Oryza sativa*)	Rice	Chimeric repressor-mediated suppression	Drought tolerance	Huang et al. (2016)
*OsNAC2*	Rice (*Oryza sativa*)	Rice	Over-expression	Salt tolerance	Shen et al. (2017)
*OsNAC5*, *OsNAC6*, *OsNAC9*, *OsNAC10*	Rice (*Oryza sativa*)	Rice	Over-expression	Drought tolerance and high grain yield	Chung et al. (2018); Fig. 5b
*OsSNAC1*	Rice (*Oryza sativa*)	Cotton (*Gossypium hirsutum*)	Over-expression	Drought and Salt tolerance	Liu et al. (2014)
*OsSNAC1*	Rice (*Oryza sativa*)	Wheat	Over-expression	Drought and Salt stresses	Saad et al. (2013)
*PgNAC21*	Pearl millet *(Pennisetum glaucum*)	Arabidopsis	Over-expression	Salinity stress tolerance	Shinde et al. (2019)
*SbSNAC1*	Sorghum (*Sorghum bicolor*)	Arabidopsis	Over-expression	Drought tolerance	Lu et al. (2013)
*SNAC3*	Rice (*Oryza sativa*)	Rice	Over-expression/RNAi	Heat and Drought tolerance	Fang et al. (2015)
*TaNAC29*	Wheat (*Triticum aestivum*)	Arabidopsis	Over-expression	Salt and Drought tolerance	Huang et al. (2015), Xu et al. (2015)
*TaNAC2a*	Wheat (*Triticum aestivum*)	Tobacco	Over-expression	Drought tolerance	Tang et al. (2012)
*TaNAC47*	Wheat (*Triticum aestivum*)	Arabidopsis	Over-expression	Salt, Drought, and Freezing stresses tolerance	Zhang et al. (2016)
*TaNAC2*	Wheat (*Triticum aestivum*)	Arabidopsis	Over-expression	Drought, Salt, and Freezing stresses tolerance	Mao et al. (2012)
*TaNAC2L*	Wheat (*Triticum aestivum*)	Arabidopsis	Over-expression	Heat tolerance	Guo et al. (2015)
*TaNAC69*	Wheat (*Triticum aestivum*)	Wheat	Over-expression	Dehydration tolerance and water use efficiency	Xue et al. (2011)
*TaRNAC1*	Wheat (*Triticum aestivum*)	Wheat	Over-expression	Enhances root length, biomass and Drought tolerance	Chen et al. (2018)
*ZmNAC1*	Maize (*Zea mays*)	Arabidopsis	Over-expression	Lateral root development	Li et al. (2012)
*ZmNAC55*	Maize (*Zea mays*)	Arabidopsis	Over-expression	Drought tolerance	Mao et al. (2016)
*ZmSNAC1*	Maize (*Zea mays*)	Arabidopsis	Over-expression	Dehydration tolerance	Lu et al. (2012)

*OsSNAC1*, a rice stress-responsive TF, was shown to ameliorate salinity and drought tolerance in wheat cultivars when over-expressed (Saad et al. 2013). Wheat plants overexpressing the *OsSNAC1* gene had elevated sensitivity to abscisic acid (ABA), which caused higher water and chlorophyll levels in their leaf tissues with an increased fresh and dry root weight. Furthermore, a higher level of *OsSNAC1* was involved in regulating the expression of abiotic stresses and ABA signaling genes such as wheat *1*-*phosphatidylinositol*-*3*-*phosphate*-*5*-*kinase*, *type 2C protein phosphatases*, *sucrose phosphate synthase*, and the regulatory components of the ABA receptor (Saad et al. 2013). Conversely, *OsSNAC1* over-expression in cotton (*Gossypium hirsutum*) plants improved drought and salt tolerance by facilitating vigorous root growth and lowering the transpiration frequency relative to non-transformed plants (Liu et al. 2014). *OsNAC6* from rice was induced by multiple stresses such as cold, abscisic acid (ABA), drought, salt, and JA, and is thus considered a redundant candidate for signals derived from abiotic as well as biotic stresses in rice (Ohnishi et al. 2005). This gene is also induced by wounding, along with other early-responsive genes (Ohnishi et al. 2005). Ochiai et al. (2011) claimed an enhanced tolerance to Boron toxicity by inhibition of the NAC-like transcription factor *BORON EXCESS TOLERANT1* (*BET1)* gene in transgenic rice. A recent example includes *ONAC066*, which is induced in response to multiple stresses: polyethylene glycol (PEG), H_2_O_2_, or salinity treatments of the *ONAC066*-overexpressing transgenic rice plants resulted in greater accumulation of soluble sugars and proline, reduction in reactive oxygen species (ROS), and water loss, thereby providing accelerated drought and oxidative resistance in rice plants (Yuan et al. 2019). Similarly, *OsNAC14* provided increased drought resistance in over-expressed rice plants during vegetative growth by repairing the damaged DNA and defense mechanism (Shim et al. 2018). Transgenic plants also had greater panicle number and a faster grain filling rate than WT (Shim et al. 2018). *OsNAC14* functions by binding to the *OsRAD51A1* promoter, a constituent of DNA repair machinery. Another case reported by Fang et al. (2015) revealed the importance of the *SNAC3* (*ONAC003*) gene in conferring heat and drought endurance through the up-regulation of high temperature responsive genes (Fig. 5a). Further, *SNAC3* overexpressing rice plants exhibited lesser electrolytic leakage, Malondialdehyde (MDA) and peroxides levels than WT at high temperatures, thereby demonstrating tolerance via ROS modulation (Fang et al. 2015). Similarly, *OsNAC5*, *6*, *9*, and *10* conferred drought endurance in over-expressed rice plants by up-regulating target genes that are responsible for altering root architecture (Chung et al. 2018, Fig. 5b). Transgenic plants also had reduced grain yield loss under drought stress compared to WT.

*TaNAC29*, a wheat (*Triticum aestivum*) NAC TF, was reported to boost tolerance against salt and drought when over-expressed in Arabidopsis plants, but compromised the flowering time (Huang et al. 2015, Fig. 5c). In another study, over-expression of *TaNAC47* in Arabidopsis caused ABA hypersensitivity resulting in the activation of a plethora of responses by altering gene expression and displayed enhanced resistance towards PEG, salinity, and freezing stresses in transgenic plants (Zhang et al. 2016, Fig. 5c). Another possible dehydration tolerance case occurred when *TaRNAC1* was over-expressed in transgenic wheat under PEG. This generated higher aboveground biomass and yield under water-deficit conditions (Chen et al. 2018). *TaNAC2L* over-expression, induced by high-temperature, stimulated heat-responsive gene expression, thereby enhancing thermotolerance in transformed Arabidopsis plants (Guo et al. 2015). Earlier, Mao et al. (2012) also reported the role of the *TaNAC2* allele in improving drought, salt, and freezing tolerance in *TaNAC2*-overexpressing Arabidopsis plants. The role of NAC family TFs in wheat is not limited to abiotic stress only. A recent report indicated that *TaNACL-D1* interaction with *TaFROG* (Fusarium Resistance Orphan Gene) can facilitate resistance to Fusarium head blight disease. Unusually, *TaNACL* harbors the *Triticeae*-specific protein in the C-terminal region (Perochon et al. 2019). As discussed in previous sections, the virtue of complexity in the C-terminus in the NAC TF genes is fascinating. Therefore, it will be interesting to see more cases of genus-specific roles of NAC members in the future.

Documentation of the role of NAC members in stress adaptation is not limited to *Triticeae. ZmNAC111*, a maize (*Zea mays*) TF, was associated with increased drought tolerance of maize seedlings and water-use efficiency (WUE), along with expression of drought-responsive genes during water deficit (Mao et al. 2015). Another report claimed the involvement of *ZmNAC55* in inducing drought resistance to overexpressing Arabidopsis plants (Mao et al. 2016). Further, *ZmNAC55* gene had multiple cis-elements related to abiotic stress acting in the promoter region.

Soybean is a crucial legume crop that is cultivated mainly to provide cooking oil and dietary protein. *GmNAC11* and *GmNAC20*, which are well-characterized genes in soybean, that are differentially expressed under many environmental stresses coupled with plant hormones (Hao et al. 2011). These genes encode proteins that localize to the cell nucleus and bind to a core DNA sequence CGT[G/A] (Hao et al. 2011). *GmNAC11* is a transcriptional activator regulating *Drought*-*responsive element*-*binding 1A* (*DREB1A*), *Early responsive to dehydration 11* (*ERD11)*, *Cold regulated 15A* (*COR15A*), *Ethylene responsive factor 5* (*ERF5)*, *Ras*-*related protein Rab18* (*RAB18*), and *Potassium channel in Arabidopsis thaliana* 2 (*KAT2*) genes, thereby building salt tolerance (Hao et al. 2011). Over-expression of *GmNAC20*, on the other hand, augments salt and freezing tolerance (Hao et al. 2011). In similar manner, Arabidopsis plants that over-expressed *GmNAC019* showed enhanced survival rates, intense antioxidant defense, lower peroxide levels and water loss under soil drying situations (Hoang et al. 2019). The transgenic plants were also found to be hypersensitive to ABA, exhibiting lower seed germination rates with fewer green cotyledons which suggested ABA-mediated regulation. Another recent report indicated the involvement of *GmSNAC49* in inducing Arabidopsis drought tolerance by up-regulating drought-responsive genes via ABA signaling period (So and Lee 2019).

Chickpea (*Cicer arietinum*), a vital legume crop in the semi-arid tropics (SAT), is naturally resistant to several abiotic stresses and suboptimal conditions. *CarNAC6* is a chickpea nuclear protein that can bind to CGT[G/A]. Over-expression of *CarNAC6* in Arabidopsis plants resulted in an enhanced drought tolerance and promoted root development under saline conditions (Liu et al. 2017). In a similar fashion, over-expression of *CarNAC4* in Arabidopsis plants led to increased expression of the stress-related genes- *Early Responsive to Dehydration 10 (ERD10)*, *Cold Regulated 15A* (*COR15A*), *Responsive to desiccation 29A* (*RD29A*), *KIN1* (*Stress*-*induced protein KIN1*), *Cold Regulated 47* (*COR47*), and *Drought*-*responsive element*-*binding A* (*DREBA*), thereby enhancing endurance to drought and saline conditions (Yu et al. 2016, Fig. 6a). *CarNAC2*, another chickpea gene encoding NAC protein (transcriptional activator), is a nuclear protein of 191 amino acids that showed enhanced resistance in transformed Arabidopsis plants when over-expressed (Yu et al. 2014). A few years ago, Peng et al. (2009) reported drought and hormone (indole-3-acetic acid and ABA) induction of the *CarNAC3* protein with a conserved NAC domain belonging to the NAP (NAC-like, activated by *APETALA3/PISTILLATA*) sub-class of the NAC superfamily. Similarly, transgenic Arabidopsis plants overexpressing drought-induced *AhNAC2* from peanuts (*Arachis hypogaea*) resulted in a greater expression of stress-related genes and a higher endurance to drought and salinity compared to the control (Liu et al. 2011). Moreover, *AhNAC2* overexpressing Arabidopsis lines were ABA hypersensitive at seed germination, stomatal closure, and root growth relative to WT plants, implying the functioning of *AhNAC2* in ABA signaling. Another report suggested that over-expression of *AtNAC2* (*ANAC092*) in groundnuts provided tolerance against salinity and drought stress, and improved yield (Patil et al. 2014). Tang et al. (2017) isolated and characterized stress-responsive *AhNAC4*, from peanut immature seeds. Peanut *AhNAC4* belonging to the ATAF subfamily was highly induced by drought. Over-expression of *AhNAC4* improved drought tolerance with an increase in stomatal closure and higher WUE in transformed tobacco (*Nicotiana tabacum*) plants compared to WT. Interestingly, when tobacco seedlings were subjected to 15 days of drought, all WT plants displayed severe wilting because of water scarcity, whereas the transgenic plants showed delayed leaf wilting. Further, the WT plants became completely desiccated, and most of them (70%) did not recover after being watered. In contrast, a higher survival ratio was seen in *AhNAC4* transgenic plants, with 90% remaining viable, indicating its importance in improving drought tolerance (Tang et al. 2017). Similarly, *MuNAC4*, a NAC TF from horse gram (*Macrotyloma uniflorum*), displayed enhanced drought tolerance in addition to proliferated lateral root growth as compared to WT when introduced into peanut plants. The imposition of long-term drought resulted in an increase in lateral roots with reduced membrane damage, increasing osmotic adjustment, and anti-oxidative enzyme regulation in transgenic peanut under stress (Pandurangaiah et al. 2014).

Pearl millet (*Pennisetum glaucum*) is an important cereal crop that is cultivated mainly in SAT for its high nutritional value. The plant, which is well known for its resistance to abiotic stress, has gained much attention since its whole-genome sequences became available (Varshney et al. 2017). *PgNAC21*, a pearl millet NAC gene, has been shown to provide salinity tolerance in transgenic Arabidopsis plant by up-regulating *COR47*, *RD20*, and *GSTF6* (*Glutathione S*-*transferase* F6) target genes (Shinde et al. 2019, Fig. 6b). Another example of SAT cereal crop *NAC* is finger millet (*Eleusine coracana*). Over-expression of finger millet *EcNAC67* in rice plants resulted in an increased root and shoot biomass, less reduction in grain yield and maintenance of higher water content leading to better survival against drought and salinity situations (Fig. 6c). Similarly, *SbSNAC1*, a member of the NAC superfamily from tropical cereal sorghum (*Sorghum bicolor*), is expressed during drought and salinity and at a relatively higher concentration in roots (Lu et al. 2013). Transgenic Arabidopsis plants that over-expressed *SbSNAC1* showed improved survival rates under drought stress accompanied by vigorous green leaves with reduced ion leakage compared with WT plants (Lu et al. 2013).

NAC TFs are undoubtedly effective as an upstream regulator of the expression of adaptive stress by downstream genes. A schematic representation of the NAC transcriptional network along with its target genes is shown in Fig. 5 as an aid to understanding their role in stress adaption in rice and wheat crops. Fine-tuning the expression of stress-specific NAC TFs is very promising for designing plant stress tolerance. However, it is also important to identify the other parallel biological processes influenced by NACs that indirectly contribute to plant adaptive responses and yield potential under negative environmental cues.

### NACs in secondary cell wall synthesis

Secondary cell walls (SCWs) are the greatest contributors to plant biomass. Secondary cell walls present in the fibers and tracheary elements of plants are comprised of cellulose, hemicelluloses and lignin. The lignocellulosic biomass represents the carbon-free raw material for generating biofuels. Thus, engineering plants with better SCW characteristics is a key approach to reducing the processing of lignocellulosic biomass. Also, plant resistance to pathogens depends on a complicated mesh of constitutive/inducible defensive barriers. In order to successfully colonize the host plant tissues, pathogens need to overcome the plant cell wall. In this context, the plant cell wall acts as a passive barrier that regulates defense processes and as a platform for signaling the molecules that activate immune responses (Miedes et al. 2014).

**Fig. 7:**
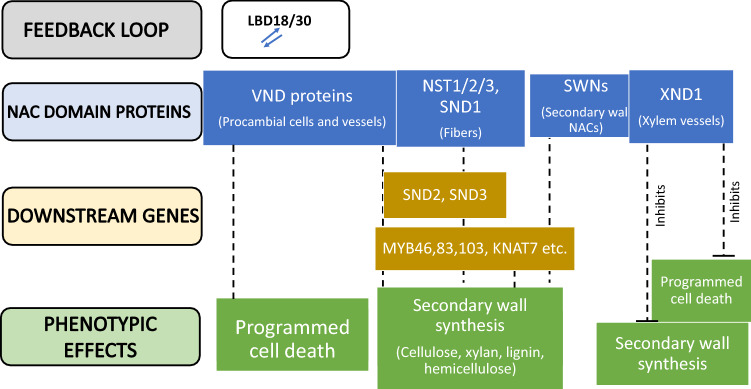
The transcriptional network regulating SCW formation by NAC domain proteins. NST1/2/3 are the master switches for controlling secondary cell wall biosynthesis in fibers. SND2/3 functions, which are downstream of NST1 and NST3, activates transcription of MYB factors and up-regulate genes associated with cellulose, xylem, hemicellulose, and lignin biosynthesis. VND6,7 and SWNs also act as regulators of SCW biosynthesis, specifically in vascular vessels. On the other hand, XND1 protein acts contrary to VND proteins by inhibiting SCW biosynthesis and PCD in xylem vessels. *SCW* secondary cell wall, *PCD* programmed cell death, *NST* NAC SECONDARY WALL THICKENING PROMOTING FACTOR, *SND2/3* SECONDARY WALL NAC DOMAIN PROTEIN, *VND* VASCULAR NAC DOMAIN PROTEIN, *SWNs* SECONDARY WALL NAC DOMAIN PROTEINS, *XND1* XYLEM NAC DOMAIN 1

NAC TFs are confirmed as mediating SCW synthesis in several species (Grover et al. 2014; Valdivia et al. 2013). A sub-group of closely related NST1 (ANAC043), NST2 (ANAC066), and NST3/SND1 (ANAC012) proteins, function as master transcriptional switches in mediating SCW formation (Mitsuda and Ohme-Takagi 2008; Singh et al. 2016), (Fig. 7). Both SND2 (SECONDARY WALL NAC DOMAIN PROTEIN2) and SND3 (SECONDARY WALL NAC DOMAIN PROTEIN3) function downstream of NST1 and NST3 (Singh et al. 2016; Zhong et al. 2008). SND2 up-regulates the genes responsible for cellulose, hemicellulose, and lignin biosynthesis and polymerization. VND6 and VND7, which are Vascular NAC Domain proteins, act as regulators of SCW biosynthesis, particularly in the xylem vessels (Kubo et al. 2005; Yamaguchi et al. 2008). However, XND1, which is a Xylem NAC Domain1 protein, acts as a negative regulator of secondary cell wall formation in xylem vessels by inhibiting VND functions to activate SCW associated gene expression.

Over-expression of *SND2* in Arabidopsis caused up-regulation of the biosynthetic genes that encode components to initiate the formation of SCW (cellulose and lignin polymerization; Hussey et al. 2011). SND2, which occupies a central role in the transcriptional regulatory network for the SCW synthesis, also upregulates the MYB103 TF and *SND1* when it is over-expressed. *SND2* over-expression also negatively influences the fiber wall deposition in Arabidopsis; in contrast, its over-expression in *Eucalyptus* caused an increase in fiber cell area (Hussey et al. 2011). Hussey et al. (2011) also highlighted the importance of determining expression thresholds for *SND2* over-expression that are optimal for an enhanced SCW deposition, since expression beyond the threshold led to co-suppression.

Rice *OsSND2*, a close homolog of *AtSND2*, was discovered to be associated with regulating SCW biosynthesis in rice and offered a scheme for engineering the green biomass production (Ye et al. 2018). *OsSND2* is primarily expressed in the internodes and the panicles, and its over-expression results in up-regulation of SCW biosynthetic genes and increased cellulose content (Ye et al. 2018). Other potential candidates include SECONDARY WALL NAC DOMAIN PROTEINs (SWNs) regulating the formation of SCW in rice (OsSWNs). They are being used for engineering the cell wall in monocotyledonous forage crops applications. For instance, the *OsSWN2S* chimeric repressor, which has a very low ability for transcriptional activation, when driven by *OsSWN1* (high transcriptional activation ability) promoter, results in a reduced cell wall thickening (sclerenchymatous cells) with low lignin and xylose contents (Yoshida et al. 2013). Similarly, Chai et al. (2015) reported that over-expression of *OsSWN1* results in SCW-related gene expression with improved lignin and reduced sugar build-up in transgenic plants. Since *OsSWN1*-like genes are highly conserved in crops such as rice, sorghum, and switchgrass, this indicates the potential of exploiting the *OsSWN1* orthologs in other crop species used as food and fodder (Chai et al. 2015).

Zhao et al. (2010) identified genes responsible for SCW biosynthesis in the model legume *M. truncatula*. Insertion of *Tnt1* retrotransposon in a NAM-like protein MtNST1 (NAC SECONDARY WALL THICKENING PROMOTING FACTOR 1) resulted in a reduced expression of cellulose, hemicellulose, and lignin biosynthetic genes. Hence, a lack of lignin in phloem fibers, decreased vascular lignin, and reduced cell wall polysaccharide content were found associated with loss of MtNST1 function (Zhao et al. 2010). Similarly, *NST1* gene in *M. truncatula* that has lost the function mutation (T94K) produced no lignification in interfascicular fibers (nst1-3 mutant), as in the case of tnt1 transposon insertion alleles (Wang et al. 2011). T94K mutation in *SND1* (Arabidopsis homolog) caused loss of target binding with the resultant incompetence to activate secondary wall synthesis genes. Moreover, *SND1* expression undergoes positive feedback control from itself, and tethers directly to a conserved motif present in its promoter region (Wang et al. 2011).

The root cap is a multilayered column consisting of parenchyma cells that lie on the top of the growing root tip and strengthen growth by taking care of the root meristem and sensing gravity in addition to rhizospheric interaction. NAC family members, viz., *SMB* (*SOMBRERO*), *BRN1* (*BEARSKIN1*), and *BRN2* (*BEARSKIN2*), along with *VND* and *NST* genes, were found to be involved in root cap maturation and showed similar phenotypic patterns when over-expressed (Bennett et al. 2010; Mitsuda et al. 2005, 2007; Zhong et al. 2006, 2007). Over-expression of NAC proteins (SMB, BRN1, BRN2) class IIB activated transcriptional pathways, resulting in secondary cell wall synthesis (SCW) by transcription of *VND*, *NST*, and root cap maturation genes (Bennett et al. 2010; Kamiya et al. 2016).

### NACs in yield potential (grain yield), seed size and biomass

Nitrogen (N) is among the primary nutrients that influence plant productivity, and limited N supply causes major constraints on crop yield (Jones et al. 2013). Well-developed roots are required for an efficient N acquisition. Nitrogen uptake, assimilation, remobilization, and storage are regulated by complex gene networks (Masclaux-Daubresse et al. 2010). In major cereal crops, N assimilation genes often colocalize with quantitative trait loci (QTLs) influencing grain yield and nitrogen use efficiency (NUE) (He et al. 2015). Several studies have indicated that expression of various NAC members was related to nutrient seed development and deficiency stress (Agarwal et al. 2011; de Zélicourt et al. 2012; Vidal et al. 2013). These yield associated NACs hold great potential as targets for crop improvement, and their functions should be explored further. *AtNAC4*, a target of the *AtAFB3* (*AUXIN SIGNALING F*-*BOX3*), has been shown to be a principal regulator of the nitrate-responsive network in Arabidopsis (Vidal et al. 2014). In a different finding, *AtNAC4* was reported to work upstream of the OBF BINDING PROTEIN4 (*AtOBP4)*, a zinc finger TF to induce nitrate response (Vidal et al. 2013). In rice, the *PS1* (prematurely senile)/*Oryza sativa* NAC-like, transcriptionally activated by *APETALA3*/*PISTILLATA* (*OsNAP*), controls N and other nutrient stockpiling in grains. *OsNAP* over-expression up-regulated the genes for various amino acids and peptide transporters and significantly promoted senescence, whereas *OsNAP* knockdown resulted in senescence delay in rice (Liang et al. 2014). Reduced *OsNAP* transcript accumulation caused delay in leaf senescence including extended grain-filling duration, leading to higher yield potential (Liang et al. 2014). *PS1* is a functional ortholog of *AtNAP* and belongs to the NAP subfamily of NAC proteins. The C-terminus of *OsNAP* functions as an activator, whereas the NAC sub-domains 3 and 4 function as a repressor (Liang et al. 2014). In another case, the over-expression of root specific *OsNAC5* in rice plants showed an increment in grain yield of 9–23% under normal conditions (Jeong et al. 2013). However, grain yield was found to be 22–63% higher under drought condition than in WT plants. Moreover, the transgenic plants developed enlarged root diameter due to an expanded stele and aerenchyma at the flowering stage, contributing to enhanced drought tolerance (Jeong et al. 2013). In addition to this, *GLP* (Germin-like protein), *PDX* (Pyridoxin biosynthesis protein), *MERI5* (Meristem protein) and *O*-*methyltransferase* were found to be up-regulated in transgenic rice. Further, *OsNAC5* also plays an essential role in loading iron to the seeds via senescence signaling (Ricachenevsky et al. 2013). In contrast to the positive up-regulation of yield by NAC members, a recent study showed over-expression of miR164b and down-regulation of *OsNAC2* in rice indicating improved plant architecture and increased grain yield/number than in WT plants (Jiang et al. 2018).

Rice *ONAC020*, *ONAC026*, and *ONAC023* genes are highly expressed during seed development (Mathew et al. 2016). *ONAC020* and *ONAC026* belong to the same phylogenetic clade as *CUC3*, an important gene in seed development (Mathew et al. 2016). *ONAC020* and *ONAC026* are closely related to *CUC3*, and all three possess typical NAC sub-domains architecture and a NAC repression domain (NARD) (Mathew et al. 2016). It is worth mentioning that expression levels for these genes varied among rice accessions with contrast in seed size. These genes regulate downstream genes to a varying magnitude due to sequence alterations in the promoter’s regions. These *ONAC020* and *ONAC026* genes have a DLN stretch (ERF-associated repression motif in plants) in the B domain. Repressor *ONAC026* dimerizes with trans-spliced forms of *ONAC020* (*ONAC020.A*, *ONAC020.B*, *ONAC020.C*), or *ONAC023* to form a heterodimer complex that localizes in the nucleus. It is most likely that the expression levels (repression/activation) of the complex may vary across the seed developmental stages. Further, these seed size-related *NAC* genes, function in seed development processes and can be utilized as possible targets for crop improvement (Mathew et al. 2016). Moreover, seed size improvement is not only an attribute of yield potential; acquiring the capability for enhanced micronutrient uptake and utilization is also very important.

**Fig. 8:**
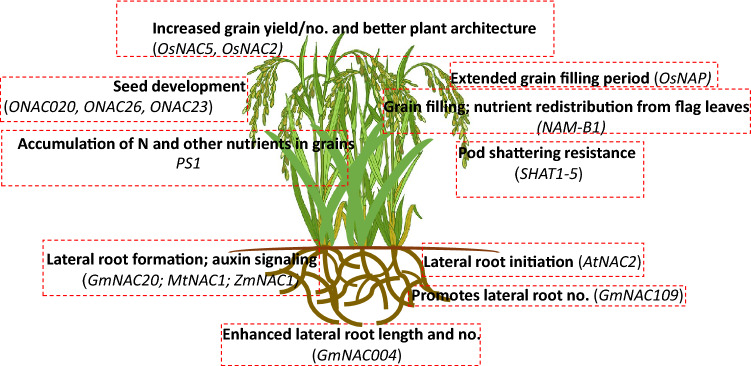
Pictorial representation of different *NAC* roles associated with lateral root development and yield related traits

Plants with stay-green phenotypes exhibit inefficient N remobilization, resulting in low harvest index, despite having the potential for higher productivity (Gregersen et al. 2008). However, plants with faster senescence exhibit an efficient N remobilization, leading to high grain protein content but with reduced grain yield (See et al. 2002). Several studies indicated that *NAC* genes are promising targets in breeding approaches for increased grain quality (Fig. 8) and nutritional potential. For instance, wheat *Gpc*-*B1* (*Grain protein content*-*B1*) encodes a NAC protein NAM-B1, with accelerated senescence in flag leaves. NAM-B1 is closely related to Arabidopsis ANAC025, ANAC018, and ANAC056 proteins. At the grain filling stage, *NAM*-*B1* (*Gpc*-*B1*) mediates nutrient redistribution from flag leaves to ears and accelerates senescence (Uauy et al. 2006). RNA interference (RNAi) mediated knockdown of *NAM*-*B1* resulted in senescence delay, leading to lower nutritional and protein contents in the grain but increased nutritional contents and residual N in the flag leaf. Similarly, *HvNAM*-*1* and *HvNAM*-2 genes, (*Gpc*-*B1* homologs) were identified in barley (*Hordeum vulgare*) (Uauy et al. 2006). Spikelet initiation and growth, along with leaf senescence subsequent to floral transition, were influenced by barley GPC locus (Lacerenza et al. 2010; Parrott et al. 2012). The near-isogenic line with high GPC expression exhibited faster development and earlier flowering. (Lacerenza et al. 2010). Additionally, several genes were up-regulated in the senescing near-isogenic barley line, and hence could be involved in senescence regulation (Jukanti and Fischer 2008; Jukanti et al. 2008). These findings signify that NAM TFs are necessary for nutrient distribution not just in wheat but in other cereal crops as well.

*Sorghum bicolor* is extensively grown for food, forage, sugar, and biomass. The sorghum Dry Stalk (D) locus contains NAC TF *SbNAC074a*, and co-localizes with sugar yield-related QTL (Xia et al. 2018). *SbNAC074a* is associated with a premature stop codon that disrupts the NAC domain in the recessive parent (dd) and is responsible for alterations in the biomass. Thus, the near-isogenic lines (NILs) of Green midrib (dd) displayed decreased stalk lignin contents but increased soluble sugar levels and grain yields under normal field situations. (Xia et al. 2018).

An important yield related *NAC* gene example is Pod shattering resistance by *SHAT1*-*5*, (*SHATTERING1*-*5). SHAT1*-*5* from soybean which causes fiber cap cell secondary walls thickening when they are over-expressed at 15-fold, thereby causing disruption of an upstream repressor (Dong et al. 2014). The critical cellular function of the shattering-resistant feature exists is the excess of lignification of fiber cap cells while the abscission layer is unaltered in the pod ventral suture. The subsequent loss of seed dispersal is a crucial agronomical trait that is a keystone feature in crop cultivation. The tissue development and differentiation control by NAC family TFs are therefore important factors that remain underexplored though they hold potential for improving future crops.

### Lateral root development regulation by NACs

Lateral root formation is a vital root developmental feature related to the phenotypic adaptation to salinity and drought stress. NAC TFs have been found to be directly linked to lateral root initiation (Fig. 8), with *AtNAC2* being perhaps the best example of this (He et al. 2005). *AtNAC2* is preferentially expressed at high levels in roots and flowers. Notably, *AtNAC2* expression is up-regulated in ethylene and auxin overexpressing mutants when exposed to salt stress (He et al. 2005). Under salt stress and heavy metal stress, lateral root development is promoted, while taproot growth is inhibited to reduce stress effects (Bhati et al. 2016; He et al. 2005). However, salt induction of *AtNAC2* expression was not linked to the ABA signaling pathway (He et al. 2005).

*AtNAC1* is also well known for its involvement in lateral root development, so its closest homolog (*MtNAC1*) was analyzed to determine its role in the lateral root formation in *M. truncatula* (D’haeseleer et al. 2011). It was found that *MtNAC1* expressed a different pattern in response to auxin than did *AtNAC1*. Plants with *MtNAC1* expression displayed no changes in the lateral root number, whereas the nodule number was reduced due to miR164 over-expression. The *NAC1* regulation by miRNA is not limited to *M. truncatula*. Maize (*Zea mays*) *ZmNAC1* (TC258020) expression is regulated by miR164 and thus influences the development of lateral roots in maize inbred lines, 87-1 and Zong3 (Li et al. 2012). Zong3 inbred line showed a 1.8-fold higher expression level of *ZmNAC1* in its roots than did the 87-1 line. Additionally, Zong3 inbred lines showed higher lateral root density than 87-1. Over-expression of *ZmNAC1* in transgenic Arabidopsis had showed enhanced lateral root growth compared to their WT (Li et al. 2012). Higher expression of mature and miR164 precursors (trans-element) was observed in 87-1 than in Zong3, which is opposite to *ZmNAC1* expression patterns, thereby contributing to differences in lateral root phenotype (Li et al. 2012).

Increase in lateral root number was reported in Arabidopsis, when they are over-expressed with the soybean *GmNAC004* gene. Basal expression levels in the *GmNAC004* homolog (*ANAC017*) were induced in roots, leaves, and flowers by water deficit stress. The *GmNAC004* gene works upstream of the key auxin regulators and increases the lateral root development in Arabidopsis through the auxin signaling pathway (Quach et al. 2014). *GmNAC20*, another soybean NAC TF, was induced to varying levels in response to plant hormones and abiotic stresses (salt and frost). Transcripts of *GmNAC20* were more abundant in cotyledons and roots. Over-expression of *GmNAC20* promoted the formation of lateral roots in transgenic Arabidopsis plants by altering genes related to auxin signaling (Hao et al. 2011). Similarly, over-expression of *GmNAC109* (*ATAF1* homolog) increased the formation of lateral roots in transgenic Arabidopsis plants by upregulating *DREB1A*, *DREB2A*, *RD29A*, *COR15A*, *AREB1* (*Abscisic acid*-*responsive element*-*binding protein 1*) and *AREB2* genes (Yang et al. 2019). The *GmNAC109*-overexpressing transgenic plants showed superior salt and drought tolerance than did WT Col-0 plants. *ABA*-*responsive genes ABI1* (*ABA insensitive 1*) and *ABI5* (*ABA insensitive 5*) were up-regulated in transformed Arabidopsis lines and were found hypersensitive to ABA (Yang et al. 2019). Downstream gene *Auxin*-*induced in root cultures 3* (*AIR3*) expression was increased, whereas *Auxin response factor* 2 (*ARF2*) showed reduced expression in these transformed lines and helped to regulate the formation of hairy root via the auxin signaling pathway (Yang et al. 2019). In summary, these findings laid the foundation for the development of soybean lines with improved tolerance to abiotic stresses via genetic modification.

### ROS signaling, leaf senescence, and programmed cell death

**Fig. 9:**
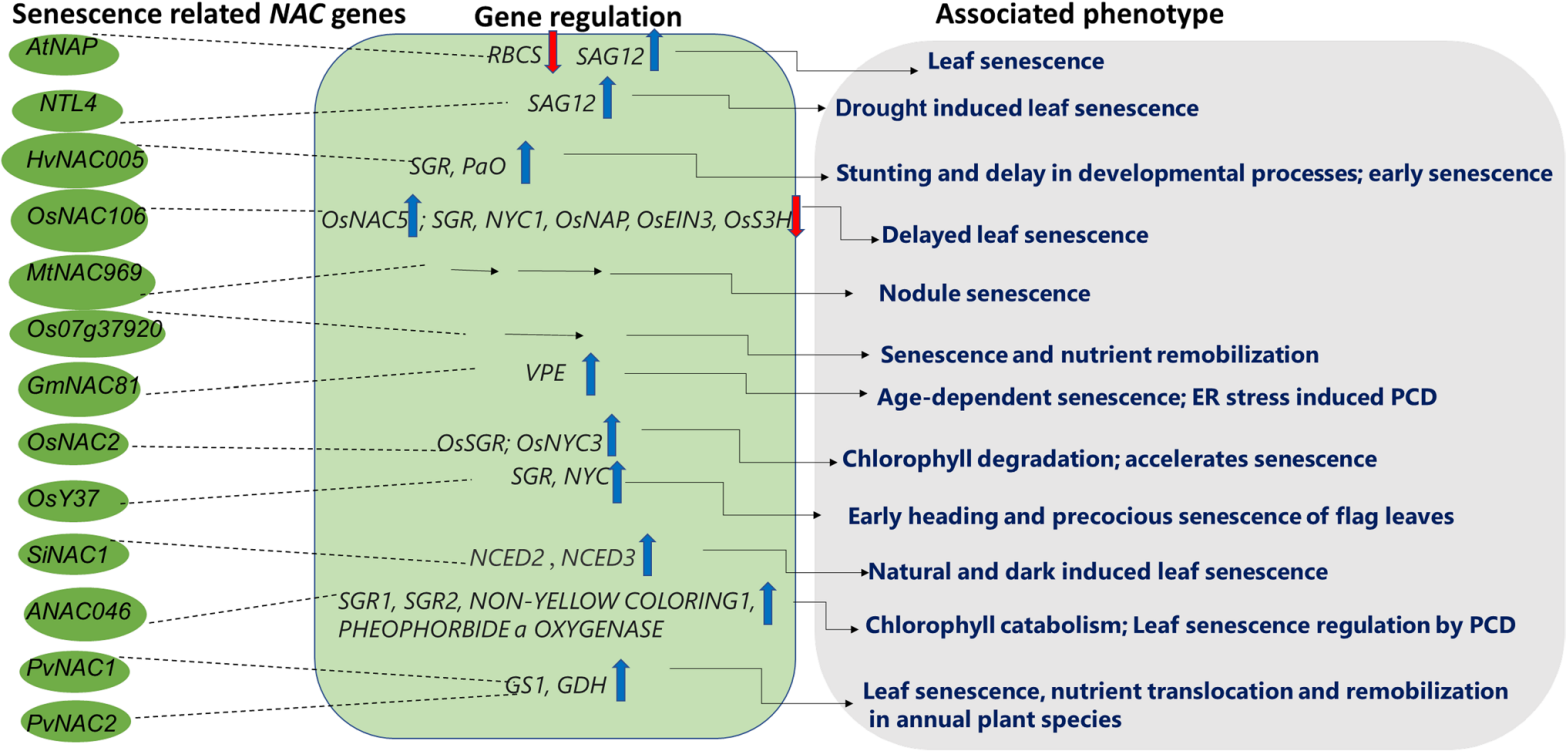
Diagram showing senescence-related NAC regulation and associated phenotypic response. Blue arrows indicate up-regulation; red arrows indicate down-regulation. *VPE* vacuolar processing enzyme, *RBCS* rubisco small subunit gene, *SAG* senescence-associated gene, *Pao* pheide a oxygenase; *NYC* non-yellow Coloring1; *NCED* nine-cis-epoxycarotenoid dioxygenase3, *GDH* glutamate dehydrogenase, *GS1* glutamine synthetase

Reactive oxygen species (ROS) and oxides such as H_2_O_2_ serve as important signal mediators that triggers plant responses against various biotic and abiotic stresses, including heavy metal stress (Bhattacharjee 2005; Davletova et al. 2005; Petrov et al. 2015). ROS signaling is a critical component of senescence and programmed cell death (PCD). Leaf senescence is necessary for the translocation of nutrients to other plant parts such as developing tissues and storage organs (Lim et al. 2007). Some of the *NAC* family genes reported to be linked with leaf senescence in various plants include *AtNAP*, *NTL4* in Arabidopsis (Guo and Gan 2006; Lee et al. 2012) and *MtNAC969* (de Zélicourt et al. 2012) in *M. truncatula*. Additionally, several other reports have emphasized the involvement of NAC proteins in leaf senescence in crops (Fig. 9). These include *SoNAP* in sugarcane (Carrillo-Bermejo et al. 2020), *GhNAP* in cotton (Fan et al. 2015), *OsNAC106* in rice (Sakuraba et al. 2015), *Os07g37920* in rice, and wheat *GPC* (Distelfeld et al. 2012, 2014). Podzimska-Sroka et al. (2015) and Kim et al. (2016) have extensively reviewed the role of NAC proteins in leaf senescence and identified various NAC-centered senescence-related gene regulatory networks (GRNs). Pimenta et al. (2016) reported *GmNAC81* mediated age-dependent senescence by endoplasmic reticulum (ER) stress-induced PCD through GmNAC81/VPE (vacuolar processing enzyme) regulatory circuit. In barley, *HvNAC005* positively controls early senescence and causes stunting and delay in developmental processes. *HvNAC005* binding to *cis*-elements of putative target genes caused up-regulation of several genes related to secondary metabolism and hormone metabolism, including those related to development, stress, and transport (Christiansen et al. 2016). Therefore, targeting *HvNAC005* in future attempts, to fine-tune gene expression related to the senescence process in barley, would be an obvious strategy for improving crop yields (Christiansen et al. 2016). Chlorophyll degradation and compromised photosynthetic efficiency are typical during leaf senescence, allowing embryos to bleach, buds to break, and fruit to ripen. In Arabidopsis, *ANAC046* has been identified to regulate the expression of Chlorophyll catabolic genes, namely, *NON*-*YELLOW COLORING1*, *STAY*-*GREEN1 (SGR1)*, *SGR2*, and *PHEOPHORBIDE a OXYGENASE* (Oda-Yamamiz et al. 2016). *ANAC046* overexpressing transgenic Arabidopsis plants showed an early-senescence phenotype and reduced chlorophyll contents compared to WT plants. This revealed that both senescence-associated genes and Chlorophyll catabolic genes were positively regulated by *ANAC046* (Oda-Yamamiz et al. 2016).

The rice NAC gene, *OsY37* (*Oryza sativa Yellow37*/*ONAC011*) accelerated heading time and promoted senescence during the terminal phase (Mannai et al. 2017). *OsY37* over-expression displayed early heading and precocious senescence in rice flag leaves, whereas knockdown of *OsY37* expression resulted in delayed heading time and leaf senescence, along with higher chlorophyll accumulation during the vegetative stage (Mannai et al. 2017). Similarly, *OsNAC2* is involved in promoting leaf senescence via ABA biosynthesis (Mao et al. 2017). Over-expression of *OsNAC2* resulted in up-regulation of *OsSGR* and *OsNYC3* genes, which are responsible for chlorophyll degradation, and hence, in an accelerated leaf senescence. Interestingly, *OsNAC2* is up-regulated by a lower ABA but down-regulated by higher ABA levels, showing that reduced expression of *OsNAC2* resulted in 10% increased grain yield in knockdown lines (Mao et al. 2017). Foxtail millet (*Setaria italica*) is another crucial food, fodder, and potential energy crop, but little is known about the functional roles of the senescence-related NAC genes of this crop. Foxtail millet *NAC1* (*SiNAC1*), an ortholog of Arabidopsis NAP (NAC-like, ACTIVATED BY AP3/PI), is involved in promoting natural and dark-induced leaf senescence by upregulating the *NCED3* gene related to ABA biosynthesis (Ren et al. 2018).

Programmed cell death (PCD) is an inherently encoded, positively controlled cellular suicide pathway that is required for the growth and survival of life forms under a compromised environment. In plants, PCD occurs during stress responses and is also involved in regular plant development. Under environmental stress, cells, tissues, or even entire organs are sacrificed to enhance the survival probabilities of the whole plant (Gadjev et al. 2008). Two NAC genes, *ANAC087* and *ANAC046* have been reported to control the expression of cell death-related genes for inducing ectopic PCD in *Arabidopsis columella* root cap cells (Huysmans et al. 2018). *ANAC087* regulates chromatin (nuclear) degradation via the nuclease BFN1 in lateral root caps. However, the genesis of cell death regulation in root caps was linked to both *ANAC087* and *ANAC046* in the course of its fall from the root tip (Huysmans et al. 2018). According to Yang et al. (2015), the *XND1*/*ANAC014* gene regulated SCW biosynthesis in Arabidopsis via PCD in xylem vessels, whereas *PvNAC1* and *PvNAC2* caused leaf senescence in annual plant species.

The findings summarized above show that NAC TFs play crucial roles in ROS signaling, leaf senescence, and PCD (Fig. 9). These reports involving various plant species have shed light on the application of NAC genes to improving plant stress responses.

## Conclusions and future prospects

Understanding the diverse mechanisms involved in stress adaptation in plants is the first step in designing suitable future-ready crops. In particular, TFs are the first line master regulators that work upstream of the gene sets responsible for the commencement of multi-omics shifts. This review highlights the *NAC* genes involved in the primary and secondary phases of stress adaptive responses in plants (Fig. 1). Various functional genomics approaches employed by these genes in model plants proved quite successful. Potentially, *NAC* genes could be great candidates for targeted engineering to develop resistance across various aspects of stress coping mechanisms, as discussed above (Table 2; Figs. 5, 6, 7, 8, 9). There remain several challenges, including the phylogenetic classification of NAC members. The C-terminus complexity and possible functional redundancy are both challenges to be overcome but both offer opportunities for exploitation. Before NAC related approaches can be integrated into breeding programs, their functional validation must be undertaken. Modern multigene/protein targeting approaches such as CRISPR-Cas9 and synthetic microProteins could help rule out the challenges of functional redundancy by identifying the NAC members with desirable functions. The growing threats of climate change-induced drought and high-temperature stresses increase the importance of identifying the specific *NAC* genes that can be used to construct stress-tolerant crops. It may also be possible to design and commercialize other transgenic plants that overexpress *NAC* genes with different stress tolerance abilities, though there have been no such reports so far. In summary, there remains great potential for the use of *NAC* genes as a biotechnological tool (as mentioned in Table 2) in years to come.
