# Correction to: The biotechnological importance of the plant-specific NAC transcription factor family in crop improvement

**DOI:** 10.1007/s10265-021-01281-9

**Published:** 2021-04-09

**Authors:** Sadhana Singh, Hiroyuki Koyama, Kaushal K. Bhati, Anshu Alok

**Affiliations:** 1 International Crops Research Institute for the Semi-Arid Tropics (ICRISAT), Patancheru, India; 2 Laboratory of Plant Cell Technology, Faculty of Applied Biological Sciences, Gifu University, Gifu, 501-1193 Japan; 3 Louvain Institute of Biomolecular Sciences, Catholic University of Louvain, Louvain-la-Neuve, Belgium; 4 Department of Biotechnology, UIET, Punjab University, Chandigarh, India

## Correction to: Journal of Plant Research 10.1007/s10265-021-01270-y

The article The biotechnological importance of the plant-specific NAC transcription factor family in crop improvement, written by Sadhana Singh, Hiroyuki Koyama, Kaushal K. Bhati and Anshu Alok, was originally published electronically on the publisher’s internet portal on February 22, 2021 without open access. With the author(s)’ decision to opt for Open Choice the copyright of the article changed on March 12, 2021 to © The Author(s) 2021 and the article is forthwith distributed under a Creative Commons Attribution 4.0 International License (https://creativecommons.org/licenses/by/4.0/), which permits use, sharing, adaptation, distribution and reproduction in any medium or format, as long as you give appropriate credit to the original author(s) and the source, provide a link to the Creative Commons license, and indicate if changes were made.

The original article has been updated.

